# Cortactin Modulates Lung Endothelial Apoptosis Induced by Cigarette Smoke

**DOI:** 10.3390/cells10112869

**Published:** 2021-10-24

**Authors:** Mounica Bandela, Eleftheria Letsiou, Viswanathan Natarajan, Lorraine B. Ware, Joe G. N. Garcia, Sunit Singla, Steven M. Dudek

**Affiliations:** 1 Department of Biomedical Engineering, College of Engineering, University of Illinois at Chicago, Chicago, IL 60607, USA; mbande4@uic.edu; 2 Division of Pulmonary, Critical Care, Sleep and Allergy, Department of Medicine, University of Illinois at Chicago, Chicago, IL 60612, USA; eletsiou@uic.edu (E.L.); visnatar@uic.edu (V.N.); ssingl6@uic.edu (S.S.); 3 Department of Pharmacology & Regenerative Medicine, University of Illinois at Chicago, Chicago, IL 60612, USA; 4 Division of Allergy, Pulmonary and Critical Care Medicine, Vanderbilt University, Nashville, TN 37232, USA; lorraine.ware@vumc.org; 5 Department of Medicine, University of Arizona, Tucson, AZ 85721, USA; skipgarcia@email.arizona.edu

**Keywords:** COPD, lung injury, e-cigarette, mitochondrial ROS, endothelium, cytoskeleton

## Abstract

Cigarette smoke (CS) is the primary cause of Chronic Obstructive Pulmonary Disease (COPD), and an important pathophysiologic event in COPD is CS-induced apoptosis in lung endothelial cells (EC). Cortactin (CTTN) is a cytoskeletal actin-binding regulatory protein with modulation by Src-mediated tyrosine phosphorylation. Based upon data demonstrating reduced *CTTN* mRNA levels in the lungs of smokers compared to non-smokers, we hypothesized a functional role for CTTN in CS-induced mitochondrial ROS generation and apoptosis in lung EC. Exposure of cultured human lung EC to CS condensate (CSC) led to the rearrangement of the actin cytoskeleton and increased CTTN tyrosine phosphorylation (within hours). Exposure to CS significantly increased EC mitochondrial ROS generation and EC apoptosis. The functional role of CTTN in these CSC-induced EC responses was explored using cortactin siRNA to reduce its expression, and by using a blocking peptide for the CTTN SH3 domain, which is critical to cytoskeletal interactions. CTTN siRNA or blockade of its SH3 domain resulted in significantly increased EC mitochondrial ROS and apoptosis and augmented CSC-induced effects. Exposure of lung EC to e-cigarette condensate demonstrated similar results, with CTTN siRNA or SH3 domain blocking peptide increasing lung EC apoptosis. These data demonstrate a novel role for CTTN in modulating lung EC apoptosis induced by CS or e-cigarettes potentially providing new insights into COPD pathogenesis.

## 1. Introduction

Cigarette smoking (CS), a leading cause of morbidity and mortality in the US, is the most significant factor for the development of Chronic Obstructive Pulmonary Disease (COPD) [1,2]. According to the Centers for Disease Control and Prevention (CDC), more than 480,000 people die from smoking-related diseases each year. Regarding the pathogenesis of CS-induced COPD, it is known that continuous exposure to nicotine and other CS-toxic substances leads to the development of chronic bronchitis and emphysema characterized by an enhanced inflammatory response, increased macrophage and neutrophil infiltration, protease-antiprotease imbalance, remodeling of the airways, and loss of elastic properties of the parenchyma [3]. Despite recent advances, there is no effective treatment available to improve or reverse smoking-related lung damage, and additional studies to elucidate CS-induced cellular mechanisms are needed. CS exposure affects not only the airways and the immune system but also alters the function of the pulmonary vasculature. Specifically, CS directly causes lung endothelial cell (EC) activation and inflammation leading to enhanced EC apoptosis and increased barrier permeability [4,5,6]. However, the signaling pathways underlying CS-induced lung EC dysfunction remain poorly understood.

Lung EC responses to multiple injurious stimuli, including CS, are mediated by cytoskeleton changes. CS exposure directly modulates EC barrier permeability, necrosis, and apoptosis through multiple signaling pathways involving RhoA, FAK, and p38 MAPK, which are all involved in cytoskeletal rearrangements [4,7,8,9]. We have previously identified cortactin (CTTN), a central regulator of the actin cytoskeleton, as an important modulator of lung EC barrier function [7,10,11]. CTTN is a major substrate for post-translational modifications (PTMs), which are dynamic and often reversible processes that regulate the functional activities of proteins within cells [10,11]. CTTN tyrosine phosphorylation on Y421, Y466, and Y486 sites by Src family kinases, ABL kinases, C-Met, FER, and Syk, regulates key cellular mechanisms such as migration, permeability, inflammation, proliferation, protrusion, and EC mechanics [11,12,13,14]. Despite these essential contributions to cellular function, the potential role of CTTN in lung EC responses to CS is unknown. Intriguingly, the gene encoding for human *CTTN* is one of the most differentially methylated in smokers vs. non-smokers [15], suggesting that its expression may be altered by cigarette exposure.

As an alternative to traditional cigarettes, e-cigarettes are gaining popularity, especially among youth. However, inhalation of e-cigarette vapors may also damage the lung tissue [16,17], and in 2019, the CDC/FDA declared an e-cigarette epidemic due to a national outbreak of e-cigarette-induced acute lung injury cases [E-cigarette or Vaping product use-associated lung injury (EVALI)] [18]. In addition, e-cigarette use is associated with an increased risk for the development of other pulmonary diseases such as COPD [19]. The underlying mechanisms by which e-cigarettes contribute to lung disease are not well understood, however, recent reports suggest that e-cigarettes play a role in inflammation, apoptosis, and tissue damage [20,21]. The effects of e-cigarettes on lung endothelial function are only now beginning to be explored.

The present study aims to explore the hypothesis that CTTN modulates the effects of CS and e-cigarettes on lung EC responses to provide novel insights into how these injurious stimuli contribute to the pathogenesis of COPD and other smoking-related disorders. Here we investigate how CTTN expression and function regulate EC responses to CS and e-cigarettes.

## 2. Materials and Methods

### 2.1. Reagents

Horseradish Peroxidase (HRP)-linked anti-mouse and anti-rabbit secondary antibodies, and anti-β actin antibodies were purchased from Santa Cruz Biotechnology, Inc. (Santa Cruz, CA, USA). Anti-CTTN antibody, phospho CTTN Y421, Y466, Y486, trypsin, Triton X-100, and Tween 20 were obtained from Sigma-Aldrich, Inc. (St. Louis, MO, USA). Antibody against PARP1 was obtained from Cell Signaling (Danvers, MA, USA). Annexin V and 7-AAD were obtained from Biolegend (San Diego, CA, USA). siRNA (control and CTTN) and DharmaFECT1 transfection reagent were purchased from Dharmacon (Horizon Inspired Cell Solutions, Lafayette, CO, USA).

### 2.2. Human Lung Tissue Specimens

Lungs from deceased organ donors that were declined for transplantation as a part of the “Beta-agonist for Oxygenation in Lung Donors” study [22] were used to obtain lung tissue specimens. Lungs were resected without perfusion and were transported on ice to the investigator’s laboratory, and portions of each lobe were immediately frozen at −80 °C in RNALater (Qiagen, Hilden, Germany) until RNA extraction. Clinical history including smoking history was obtained from the donor’s medical record. Specifically, four lung samples were obtained from chronic smokers, with smoking “pack year” histories of 50, 15, 10, and an unknown number of pack years.

### 2.3. Cell Culture

Human pulmonary artery endothelial cells (HPAECs) were purchased from Lonza (Walkersville, MD, USA) and cultured in Endothelial Cell Growth Medium-2 (EGM-2) (Lonza) supplemented with 10% fetal bovine serum (FBS) (Sigma, St Louis, MO, USA). Cells were maintained at 37 °C in a 5% CO_2_ incubator and used at passages 6–8 for all experiments. Cells were starved for 2 h in 2% FBS media prior to treatments.

### 2.4. Cigarette Smoke Condensate (CSC)

CSC was prepared by bubbling smoke from six cigarettes [Research-grade cigarettes (3R4F); Kentucky Tobacco Research and Development Center at the University of Kentucky] through 100 mL of FCS-free cell culture medium at a constant airflow. The smoked medium was then sterile filtered through a 0.20-micrometer filter (Minisart; Satorius Stedim Biotech, Göttingen, Germany), aliquoted, and stored at −20 °C. This served as the CSC stock solution (40 mg/mL concentration). For treatment, cells were exposed to 40 µg/mL of CSC in 2% EGM-2 media, and the control cells received the equivalent volume of vehicle (10% DMSO).

### 2.5. E-Cigarette

“JUUL” E-cigarette obtained commercially contains nicotine, propylene glycol, glycerin, and benzoic acid. 5.0% nicotine and the unflavored pod were used for our experiments. Cells were treated with 50 µg/mL of e-cigarette liquid for 24 h.

### 2.6. siRNA Transfection

HPAECs were transfected with scrambled RNA or CTTN siRNA (100 nM) using the DharmaFECT 1 transfection reagent. 48 h after transfection EC were challenged with CSC or e-cigarette. Transfection efficiency was determined by western blotting. 

### 2.7. Blocking Peptide Experiments

Myristoylated VDKPPVPPKPKMKPIV sequence comprising the cortactin SH3 blocking peptide (CBP) and scrambled control peptide were synthesized by the Genome Research Core of the Research Resources Center (RRC) of UIC. The efficacy of CBP in blocking interactions of the cortactin SH3 domain has been described previously [23]. HPAECs were pretreated with control or CBP, 100 µM for 45 min, followed by exposure to CSC (40 µg/mL, 24 h).

### 2.8. Determination of Apoptosis by Flow Cytometry

Flow cytometry was used to determine the levels of apoptosis as modified from previous reports [24]. Cells were stained with Annexin V and 7-AAD, according to the manufacturer’s instructions. Data were acquired using an LSR Fortessa (BD Biosciences, San Diego, CA, USA) flow cytometer and analyzed using the FCS express 6 flow cytometry (De Novo) software. A floating gating strategy was employed and was set based on unstained and single-stained populations.

### 2.9. RNA Isolation and Quantitative Real-Time PCR Analysis (qPCR)

RNA was isolated from human lung tissues (smokers/non-smokers) according to the manufacturer′s protocol. RNA was reverse transcribed using a cDNA synthesis kit (Bio-Rad, Hercules, CA, USA). qPCR was performed using the cDNA mixed with iQ SYBR Green Supermix (Life Technologies, Grand Island, NY, USA).

The primer sets used for amplification are the following:

*CTTN*, forward primer: 5′-GGTGTGGAACAAGACCGAAT-3′, reverse primer: 5′-GGCATGCTTCTCAGTCTTCC-3′; the housekeeping gene *18S RNA* served as an internal control, and all samples were run in triplicates.

### 2.10. Western Blotting

Cell lysates were prepared using RIPA buffer containing protease and phosphatase inhibitors. Samples were sonicated and centrifuged at 10,000× *g* at 4 °C for 10 min. The supernatants were collected, and protein concentration was determined using the BCA protein assay (Pierce Chemical, Rockford, IL, USA). Cell lysates were then mixed with 6X Laemmli buffer (Boston Bioproducts, Ashland, MA, USA) and boiled for 5 min. Samples (30 µg) were subjected to SDS-gel electrophoresis and then transferred to nitrocellulose membranes (Bio-Rad, Hercules, CA, USA). Membranes were incubated for 1 h at room temperature in blocking buffer (Tris-buffered saline with 0.05% Tween-20, TBST) supplemented with 1% bovine serum albumin (BSA) and then incubated with the indicated primary antibodies overnight at 4 °C. After washing with TBST, the membranes were incubated for 1 h with the secondary antibody in 1% BSA-TBST. The membranes were washed with TBST, and the bands were detected using Pierce ECL (ThermoFisher, Wilmington, DE, USA) or Amersham ECL Prime (Cytiva) followed by exposure to blue-light–sensitive film Hyper-film (Amersham Biosciences UK Limited, Little Chalfont, UK). Anti-β-actin antibody was used to verify equal protein loading. The relative intensities of protein bands were quantified by densitometry using ImageJ software (NIH, Bethesda, MD, USA). Results were expressed as a ratio of specific protein signal to β-actin.

### 2.11. Immunofluorescence Microscopy

HPAECs were grown on 8 well glass chamber slides to 80–90% confluence in EGM-2 medium. After indicated treatments, cells were fixed with 3.7% paraformaldehyde for 10 min followed by three washes with PBS. The cells were then permeabilized with 0.25% Triton X 100 for 5 min and rinsed with PBS for 5 min followed by incubation in blocking buffer (1% BSA-PBS) for 1 h. Cells were then incubated with cortactin antibody for 1 h, washed with PBS, and then incubated with secondary antibody-Alexa Fluor 488 and Alexa 594-Phalloidin (F-actin staining) for 1 h. After washing for at least four times, the coverslips were mounted with profound gold DAPI (Invitrogen, Green Island, NY, USA). Images were taken using a Zeiss confocal microscope at 40× magnification.

### 2.12. Mitochondrial ROS Generation

Mitochondrial superoxide generation in HPAECs upon CSC challenge was determined using the MitoSOX^TM^ Red Mitochondrial Superoxide Indicator (Invitrogen, Green Island, NY, USA), according to the manufacturer’s protocol. Briefly, the cells were loaded with 5 µM MitoSOX reagent for 15 min and washed twice in phenol red-free media. Live-cell imaging was performed at 37 °C using a Zeiss confocal microscope at 40× magnification.

### 2.13. Statistical Analysis

All data are expressed as mean ± SEM from at least three independent experiments. Statistical analysis was performed using the GraphPad Prism 8 software. Student’s t-test or two-way ANOVA (Tukey’s or Dunnett’s post hoc tests) were used to compare two or more groups respectively. Values of * *p* < 0.05 were considered statistically significant.

## 3. Results

### 3.1. Cortactin mRNA Levels Are Decreased in Human Lung Tissues from Smokers Compared to Non-Smokers

**Figure 1:**
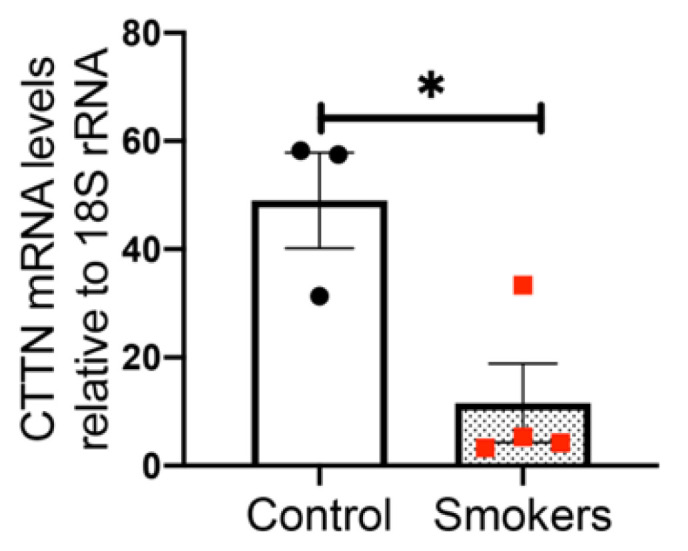
Cortactin mRNA levels are decreased in human lung tissues from smokers compared to non-smokers. Cortactin (CTTN) mRNA levels were analyzed by qPCR in human lung tissues from current versus never/former smokers. The bar graph depicts fold-changes in CTTN mRNA expression normalized to the housekeeping gene, 18S rRNA. N = 3–4, * *p* < 0.05.

Given that the gene encoding for human *CTTN* is one of the most differentially methylated in smokers vs. non-smokers [15], we analyzed human lung tissue RNA samples from donors with a history of regular cigarette smoking. These samples were assessed for *CTTN* mRNA levels by RT-PCR and compared to those from former or never smokers. We observed a significant ~80% decrease in *CTTN* expression in lung tissues derived from smokers compared to non-smokers (Figure 1).

### 3.2. Cigarette Smoke Condensate Induces Cytoskeletal Rearrangement in Human Lung Endothelial Cells

**Figure 2:**
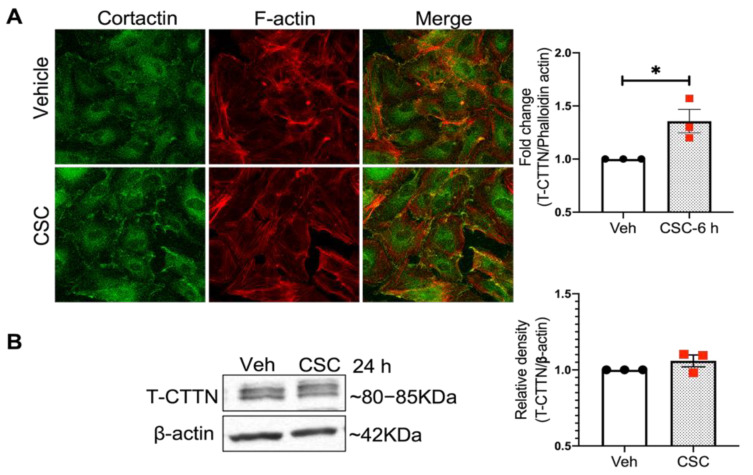
Cigarette smoke condensate induces cytoskeletal rearrangement in human lung endothelial cells. (**A**) HPAECs were treated with vehicle (DMSO) or CSC (40 µg/mL) for 6 h, fixed and subjected to immunofluorescence analysis. Confocal images were taken at 40× after staining with Alexa488 -CTTN (green), Alex 594- Phalloidin (F-actin staining, red), and DAPI (nucleus staining, blue). F-actin and total cortactin (T-CTTN) overlap was quantified. (**B**) HPAECs were treated with CSC (40 µg/mL, 24 h) and cell lysates were subjected to western blotting analysis for T-CTTN protein expression. Shown are representative blots and densitometry analysis. N = 3, * *p* < 0.05.

Next, we employed CS condensate (CSC), a well-characterized and commonly used stimulus to model CS exposure in vitro, to determine its effects on CTTN and actin cytoskeletal structure in cultured human lung EC [25]. Immunofluorescence imaging (Figure 2A) demonstrates that after CSC stimulation there is a redistribution of actin and cortactin in HPAEC that results in a significant increase in the colocalization of these proteins. Under these experimental conditions, CTTN protein expression levels are not altered by CSC exposure (Figure 2B).

### 3.3. CSC Induces CTTN Tyrosine Phosphorylation in Human Lung EC

**Figure 3:**
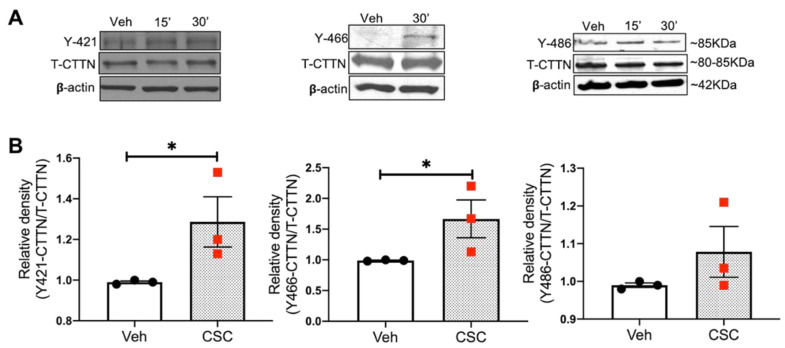
CSC induces CTTN tyrosine phosphorylation in human lung endothelial cells. HPAECs were treated with CSC (40 μg/mL) for 15–30 min. Tyrosine phosphorylation of CTTN was assessed at Y421/466/486 sites using phospho-specific antibodies for each site. (**A**) Representative blots of CTTN Y421/466/486 phosphorylation in cell lysates upon CSC challenge. (**B**) quantification of Y421/466/486 by densitometric analysis at 30 min for all 3 sites; data were normalized to total CTTN and pooled from 3 independent experiments. * *p* < 0.05.

A key posttranslational modification (PTM) of CTTN is tyrosine phosphorylation by Src, Abl, and other kinases that regulates multiple aspects of cytoskeletal rearrangement, EC permeability, cell motility, and invasion [11]. In our present study, we explored the effect of CSC on CTTN tyrosine phosphorylation at the important Y421/Y466/Y486 sites in lung EC as potential signaling events [13,14]. As shown in Figure 3, upon CSC challenge, CTTN phosphorylation is increased within 30 min at all three sites (Y421/466/486) compared to unstimulated control EC.

### 3.4. Inhibition of CTTN Expression or Its SH3 Domain Augments CSC-Induced MitoROS in Lung EC

**Figure 4:**
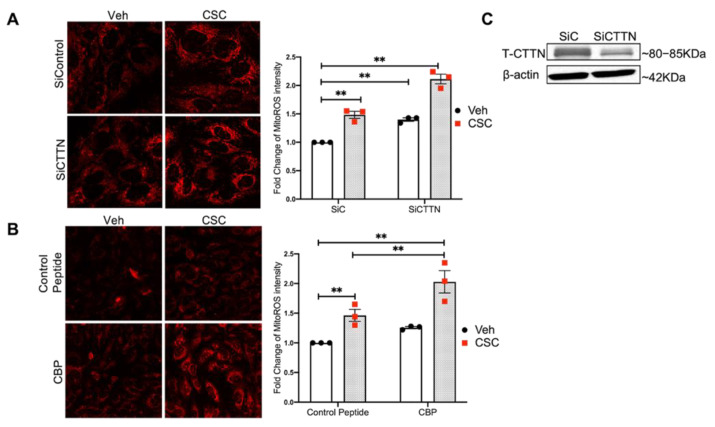
Inhibition of CTTN expression or SH3 domain blockade augments CSC-induced MitoROS in lung EC. (**A**) HPAECs were transfected with siRNA (control or CTTN) for 48 h followed by CSC (40 µg/mL) for 2 h. (**B**) HPAECs were pre-treated with peptide control or CTTN blocking peptide (CBP) for 45 min followed by CSC challenge for 2 h. Mitochondrial superoxide production was assayed by the MitoSOX TM Red reagent. Shown are the representative confocal images of mitoROS staining (**A**,**B**, left images). Quantification of ROS intensity was performed in 30 cells from 5–8 different fields for three independent experiments. ** *p* < 0.01. (**C**) Representative western blot demonstrating reduced CTTN expression in lung EC after siRNA transfection.

Mitochondrial oxidative stress plays a key role in CS-induced pulmonary disorders and is a predisposing factor in the pathogenesis of COPD [26,27]. Therefore, we next explored the role of CTTN in oxidative stress induced by CSC [28,29,30]. Using the well-established MitoSOX assay [31,32], we found that mitoROS production in lung EC is significantly increased (~1.5 fold) after CSC exposure compared to control (Figure 4A). To further elucidate the role of CTTN in this CS-induced response, *CTTN* expression was downregulated by siRNA, which resulted in a 55–75% reduction of its expression in lung EC (Figure 4C). Interestingly, *CTTN* silenced EC produced more mitoROS both at baseline and after exposure to CSC (Figure 4A). These data suggest a novel role for CTTN expression in mediating CSC-induced mitoROS production.

To expand upon these observations, we next treated lung EC with a peptide that blocks protein-protein interactions with the CTTN SH3 domain (cortactin blocking peptide; CBP). The SH3 domain of CTTN interacts with multiple proteins such as dynamin, WASP, nmMLCK, etc. that are involved in regulating multiple EC functions [33,34,35]. In the current study, CBP treatment enhanced mitoROS production at baseline and after CSC (Figure 4B), similar to cells treated with si*CTTN*. These data suggest that CTTN expression or its downstream signaling interactions are involved in mitoROS production after CSC.

### 3.5. CSC Induces Apoptosis in Lung EC

**Figure 5:**
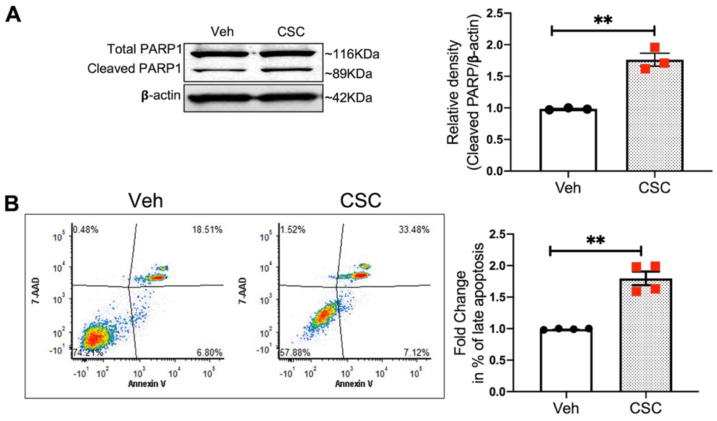
CSC induces apoptosis in lung EC. HPAECs were treated with CSC (40 µg/mL) for 24 h. Apoptosis was assessed by cleavage of apoptotic marker PARP1 and by flow cytometry of Annexin-V/7-AAD double-positive cells (% late apoptotic cells) (**A**) protein expression of cleaved PARP1 in cell lysates upon CSC challenge. Shown is a representative blot from three independent experiments and the quantification of cleaved PARP1 by densitometry. Data were normalized to β-actin. (**B**) HPAECs challenged with CSC were analyzed by flow cytometry for apoptosis. Shown are representative dot plots of cells stained with Annexin V and 7-AAD (left) and bar graphs depicting normalized percentages of late apoptotic cells under each condition (right). ** *p* < 0.01.

Pulmonary EC plays an important role in maintaining vascular homeostasis, while CS exposure causes cellular injury and leads to lung tissue damage over time. Previous studies have demonstrated that CS induces apoptosis in endothelium in vitro [4,5]. Consistent with these reports, here we demonstrate that CSC exposure (24 h) significantly increases apoptosis in human lung EC as assessed by two complementary indices: 1) western blot levels of PARP cleavage, which is an apoptotic marker; and 2) the percentage of annexin-V/7-AAD double-positive cells (late apoptotic) as determined by flow cytometry [24]. Our data demonstrate that CSC induces ~1.8-fold increase in cleaved PARP1 expression (Figure 5A), and a ~1.9-fold increase in the percentage of apoptotic cells (Figure 5B) compared to control.

### 3.6. CTTN Expression Regulates CSC-Induced Apoptosis in Lung EC

**Figure 6:**
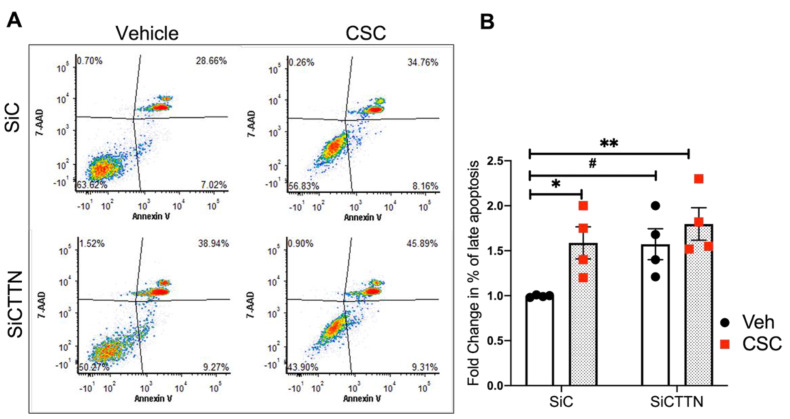
CTTN expression regulates CSC-induced apoptosis in lung EC. HPAECs were transfected with siRNA (control or CTTN) before CSC challenge (40 μg/mL, 24 h). Apoptosis was assessed by flow cytometry. (**A**) Shown are representative dot plots of cells stained with Annexin V and 7-AAD, and (**B**) Bar graphs represent the percentage of double-positive cells (late apoptosis) pooled from 4 independent experiments. * *p* < 0.05, ** *p* < 0.01, *^#^ p* = 0.053.

CTTN regulates endothelial permeability and other aspects of EC function [36], with some prior reports also suggesting a role for CTTN in apoptosis [37]. However, no prior studies have explored a potential role for CTTN in mediating EC apoptosis induced by CS. Here we explored how CTTN expression affects lung EC apoptosis at baseline and after CSC exposure. As assessed by flow cytometry, apoptosis is increased both at baseline (~1.5 fold) and after CSC (~1.9 fold) in CTTN-silenced EC compared to control siRNA-exposed cells (Figure 6A,B). These results suggest a protective role of cortactin in minimizing apoptosis in lung EC.

### 3.7. CTTN SH3 Domain Interactions Regulate CSC-Induced Apoptosis in Lung EC

**Figure 7:**
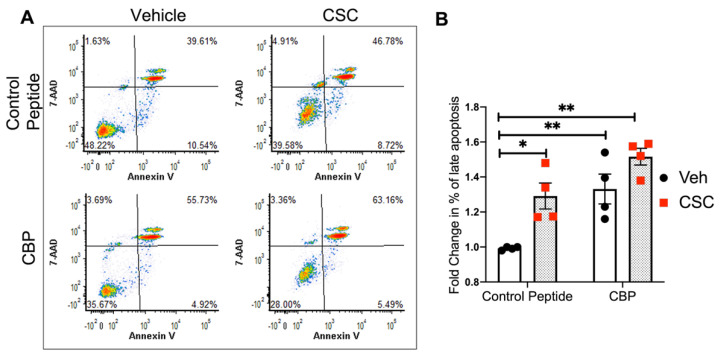
Blocking the SH3 domain of CTTN accentuates CSC-induced apoptosis in lung EC. HPAECs were pre-treated with peptide control or CTTN blocking peptide (CBP) for 45 min followed by CSC challenge (40 μg/mL, 24 h). Apoptosis was assessed by flow cytometry. (**A**) Shown are representative dot plots of cells stained with Annexin V and 7-AAD, and (**B**) Bar graphs represent the percentage of double-positive cells (late apoptosis) pooled from 4 independent experiments. * *p* < 0.05, ** *p* < 0.01.

In additional experiments, lung ECs were pretreated with CBP or control peptide for 45 min, followed by CSC challenge for 24 h. Flow cytometry assessment of apoptosis demonstrated an increase in the percentage of apoptotic cells after CBP in the vehicle and CSC-stimulated EC compared to control peptide (Figure 7A,B), suggesting that SH3-mediated interactions of CTTN are functionally involved in lung EC apoptosis.

### 3.8. CTTN Expression Regulates E-Cigarette-Induced Apoptosis in Lung EC

**Figure 8:**
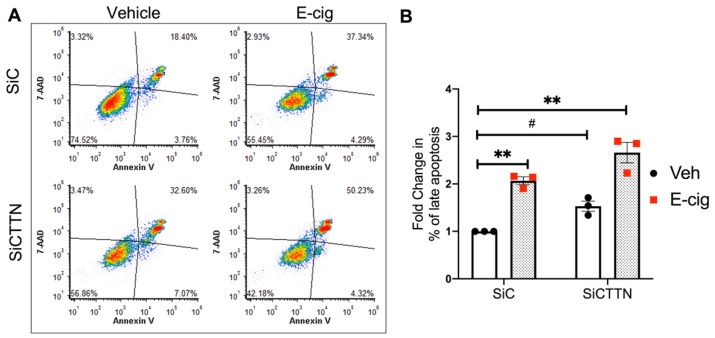
CTTN expression regulates e-cigarette-induced apoptosis in lung EC. HPAECs were transfected with siRNA (control or CTTN) before e-cigarette (E-cig) challenge (50 μg/mL, 24 h). Apoptosis was assessed by flow cytometry. (**A**) Shown are representative dot plots of cells stained with Annexin V and 7-AAD, and (**B**) Bar graphs represent the normalized percentage of double-positive cells (late apoptosis) pooled from 3 independent experiments. ** *p* < 0.01, *^#^ p* = 0.07.

Based on recent in vitro and in vivo studies, e-cigarettes also cause adverse effects in cultured cells [38]. To evaluate the role of CTTN in e-cigarette-induced apoptosis, HPAEC were treated with *CTTN* siRNA and then exposed to commercially available e-cigarette extract for 24 h. Similar to CSC, e-cigarette exposure caused an increase in the percentage annexin V/7-AAD double-positive cells, suggesting induction of apoptosis (Figure 8A,B). In CTTN-silenced EC the percentage of apoptotic cells was significantly increased at baseline and after e-cigarette exposure to ~1.5 and ~2.5 fold, respectively, compared to control cells (Figure 8A,B).

### 3.9. CTTN SH3 Domain Interactions Regulate E-Cigarette-Induced Apoptosis in Lung EC

**Figure 9:**
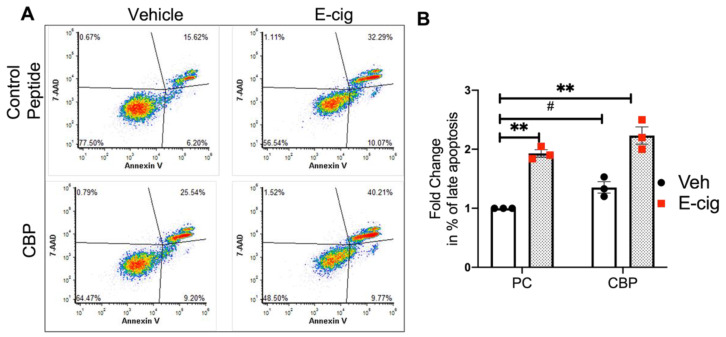
Blocking the SH3 domain of CTTN accentuates e-cigarette-induced apoptosis in lung EC. HPAECs were pre-treated with peptide control or CTTN blocking peptide (CBP) for 45 min followed by e-cigarette (E-cig) challenge (50 μg/mL, 24 h). Apoptosis was assessed by flow cytometry (**A**) Shown are representative dot plots of cells stained with Annexin V and 7-AAD and (**B**) Bar graphs represent the normalized percentage of double-positive cells (late apoptosis) pooled from 3 independent experiments. ** *p* < 0.01, *^#^ p* = 0.06.

Inhibition of CTTN SH3 domain interactions after treating EC with CBP increased the percentage of apoptotic cells by ~1.5-fold at baseline and by 2-fold after e-cigarette stimulation (Figure 9A,B). Taken together, these data further support a role for CTTN downstream signaling in regulating EC apoptosis induced by an e-cigarette.

## 4. Discussion

**Figure 10:**
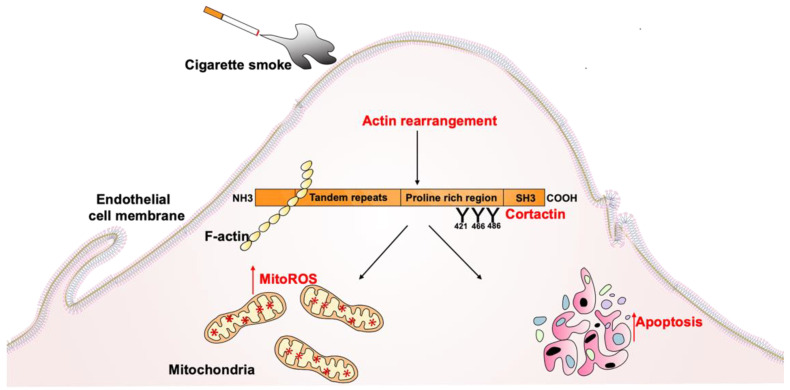
Schema illustrating the mechanistic role of CTTN in mitoROS and lung endothelial apoptosis. CTTN contributes to cigarette smoke-induced lung injury by causing rearrangement of actin and activating Src-mediated phosphorylation at the major sites 421, 466, and 486. Cigarette smoke induces mitochondrial ROS production and lung endothelial apoptosis. In vitro studies showed altering CTTN expression or blocking its SH3 interaction domain enhanced mitochondrial ROS production and endothelial apoptosis. Here, we explore the undescribed mechanism of CTTN in mtROS induced apoptosis.

The major risk factors associated with the development and progression of CS-induced lung disease are the duration and magnitude of tobacco smoking, environmental exposures, infections, and other genetic risk factors [39]. CS-induced lung damage causes airway inflammation, which is characterized by functional and structural changes in the cells of the respiratory system. CS has toxic effects on the extracellular matrix, pulmonary epithelium, and lung endothelium, resulting in vascular inflammation, increased oxidative stress, and altered cell homeostasis [40]. Adding to this prior literature, the major findings of our current study are the following: (i) Gene expression of *CTTN* is reduced in human lung tissues from smokers compared to nonsmokers; (ii) CSC caused actin cytoskeleton rearrangement in cultured human lung ECs; (iii) CSC and e-cigarette extract induce apoptosis in lung EC; (iv) CSC induces mitochondrial ROS, while the reduction in *CTTN* expression by siRNA or blocking of CTTN SH3 domain accentuated mitochondrial superoxide production; (v) Reduction in *CTTN* expression by siRNA or blocking of its SH3 domain enhanced apoptosis induced by CSC or e-cigarettes in lung EC (Figure 10).

The novel focus on cortactin in this study is based upon several prior observations. Chronic cigarette exposure may alter CTTN expression and/or function through multiple effects. It is intriguing to speculate that these effects may include DNA methylation, which is a critical epigenetic regulator of gene expression, and abnormalities in methylation status contribute to human diseases [41,42]. A recent report identified the *CTTN* gene as one of the most highly altered in terms of methylation status in smokers, suggesting a potential role for CTTN in CS-induced pulmonary diseases [15]. DNA methylation status was not addressed in our current study and remains a potential mechanism for further exploration. Regarding other possible effects of CS, it is known to alter actin cytoskeletal dynamics in human EC [43] and induce apoptosis [44,45,46]. These prior studies led us to hypothesize that the key cytoskeletal protein, CTTN, plays a functional role in mediating CS-induced cytoskeletal changes and apoptosis in lung EC.

Consistent with this hypothesis, in our current study, *CTTN* gene expression is decreased in the lungs of human smokers compared to non-smokers (Figure 1). Furthermore, in the human lung, EC CSC induces cytoskeletal rearrangement, increases CTTN interaction with F-actin, and stimulates CTTN tyrosine phosphorylation at the major regulatory sites 421, 466, and 486 (Figure 2 and Figure 3), further suggesting an important role for CTTN in CS effects. In addition, CS is known to increase the production of ROS that leads to exacerbated cellular injury [30]. CS-induced ROS production affects the morphology and function of lung epithelium and endothelium, resulting in disruption of adherens junctions, decreased Nrf2 activity, and reduced E-cadherin expression [47]. Mitochondria play a key role in activating multiple signaling pathways and maintaining baseline ROS production, while alteration in mitochondrial dynamics such as fission and fusion due to chronic exposure of CSC causes an imbalance in the normal cellular functions of proliferation and apoptosis [48]. Under some conditions, mitochondria associate with CTTN, suggesting a potential role in the assembly of F-actin during apoptosis induced by mitochondrial fission [49]. Therefore, we examined mitochondrial dysfunction by exploring the role of mitoROS in the context of CSC-induced lung dysfunction. In our study, CSC increases oxidative stress in human lung EC, which is significantly exacerbated when CTTN levels are reduced by siRNA (Figure 4), supporting a functional role for CTTN in modulating oxidative stress in these cells.

As noted above, the carboxy-terminal SH3 domain of CTTN mediates protein-protein interactions and regulates various cellular processes [33,35,50]. Blocking interactions at this SH3 domain increases mitoROS production at baseline and after CSC (Figure 4), suggesting a novel role for interactions at the CTTN SH3 domain in regulating CS-induced mitochondrial oxidative stress [28,29,48]. Several CTTN SH3 binding partners are associated with the regulation of the apoptotic pathway and could participate in mediating this effect. These include SHANK2, Arp2/3, and Dynamin 2 [51,52,53]. Another important cytoskeletal binding partner for CTTN at its SH3 domain is nmMLCK [23,35]. nmMLCK dysregulation causes caspase-dependent pulmonary EC apoptosis upon TNF-α stimulation [54]. Like nmMLCK [54], CTTN contains two clusters of caspase cleavage sites, defining CTTN as a substrate of caspase 3 [37]. The SH3 domain of CTTN was degraded in a caspase-dependent manner, dissociating actin binding and SH3 domain thereby affecting cell signaling during apoptosis [37]. These authors further identified the presence of a caspase 3 cleavage site in the actin-binding domain of CTTN and reported that CTTN degradation is associated with executionary apoptotic caspase during influenza infection [55,56]. In gastric cancer cells, overexpressing CTTN resulted in an increased percentage of apoptosis, increased pro-apoptotic marker Bax, and decreased anti-apoptotic marker Bcl-2 [57]. In contrast, our data demonstrate that reduction in CTTN expression or inhibition of its SH3 domain increases apoptosis in human lung EC at baseline and in response to CSC or e-cigarettes (Figure 6, Figure 7, Figure 8 and Figure 9). Differences in cell type, pathophysiologic state (e.g., cancer versus CSC exposure), or other experimental factors between these studies may account for this apparent discrepancy regarding the effect of CTTN expression level and apoptosis rates. Given these observations, it is interesting to speculate that CTTN may function to modulate the level of apoptosis either up or down depending on cellular status. Additional work is needed to explore this hypothesis.

A complex mixture of chemicals in CS contributes to the development of COPD, which is characterized by the destruction of the endothelium, epithelium, connective tissue, and alveoli. Nicotine is a major component present in cigarettes and e-cigarettes which leads to ROS production and mediates apoptosis [58,59,60,61]. Other active components reportedly involved in CS-induced ROS production and apoptosis are formaldehyde, benzene, and isoprene [46,62], while the active components of e-cigarette involved in pulmonary dysfunction include propylene glycol and glycerin [63,64]. Additional work is needed to explore the effect of other active components of cigarette smoke and e-cigarette in lung endothelial apoptosis.

Because cigarette smoking causes the majority of COPD morbidity and mortality, while quitting smoking and the use of alternate strategies lower risk and increase life expectancy [65], some health care experts have advocated for e-cigarettes as a safer alternative [66,67]. However, inhalation of nicotine-containing e-cigarettes leads to an increase in cytokine levels, protease expression, and airway enlargement, similar to the pathophysiologic effects of CS [60,68,69]. A study in human pluripotent stem cells derived from EC showed that nicotine-containing e-cigarettes increase mucin production, decrease cell viability, induce oxidative stress, increase caspase 3/7 activity, and impair migration, demonstrating that e-cigarettes lead to lung EC dysfunction [70,71]. In human microvascular EC, e-cigarettes induced lung inflammation and oxidative stress, thereby causing a loss in endothelial permeability associated with phosphorylation of MLC and Rho kinase [70]. Both e-cigarettes and CSC induce ROS, DNA damage, and vascular apoptosis, while the antioxidant N-acetyl cysteine prevented e-cigarette/CSC-induced cell death [21,68]. E-cigarettes induce a caspase-mediated apoptotic pathway in human epithelial cells [72]. In our current study, reduction in CTTN expression via siRNA or blockage of its SH3 domain resulted in an increase in the percentage of apoptotic cells after e-cigarette exposure (Figure 8 and Figure 9), similar to observations made with CSC (Figure 6 and Figure 7). These data provide novel mechanistic insights and add to the growing body of literature concerning the potentially harmful effects of e-cigarette exposure on lung EC.

There are some limitations in the present study. First, we analyzed CTTN expression in only a small number of human lung tissue samples, and these initial observations will need to be confirmed with additional patient samples in the future. However, these data are consistent with a recent study demonstrating that the *CTTN* gene is one of the most highly methylated in cigarette smokers, suggesting that CTTN pulmonary expression may be downregulated in smokers [15]. Further confirmatory expression studies in a larger patient cohort and assessment of CTTN DNA methylation status are needed. Second, our observations regarding a novel role for CTTN in regulating CSC/e-cigarette-induced apoptosis in lung ECs will require further exploration in vivo to determine pathophysiological relevance in whole organisms. Third, commercially available e-cigarette liquid was used for our study, and future studies should focus on vaporing e-liquid to better mimic the real-life exposure scenario. Fourth, important functional heterogeneity can occur among endothelial cells located in different vascular beds, and our present study characterizes responses only in macrovascular lung EC (HPAECs). However, we have observed similar qualitative responses to CSC in some preliminary experiments with microvascular cells (HLMVECs), which will be further characterized in future work. Fifth, additional gain-of-function studies will be useful to confirm the potential of CTTN expression to attenuate CSC-induced apoptosis and help determine the additional mechanism(s) by which CTTN regulates lung EC apoptosis pathways in response to CSC/e-cigarettes.

## 5. Conclusions

In summary, here we demonstrate that CS alters the cytoskeletal structure and CTTN tyrosine phosphorylation in lung EC, increases mitoROS, and induces apoptosis. Reduction in CTTN expression, or inhibition of its interactions with other proteins via its SH3 domain, exacerbates CS-induced mitochondrial ROS and apoptosis in lung EC. Our results suggest that this series of events occur in chronological order after CSC exposure. Upon exposure to CSC, rapid phosphorylation of CTTN occurs within minutes in lung EC, followed by cytoskeletal rearrangements and associated ROS production, and then finally induction of apoptosis as a downstream functional effect. These data reveal a previously undescribed important role for CTTN in regulating lung endothelial functions in response to CS and suggest CTTN as a potential new mediator in vascular dysfunction underlying COPD. Therefore, modulating CTTN activity during CSC-induced lung endothelial apoptosis may have important functional effects during the key step in the pathogenesis of COPD and other smoking-related pulmonary disorders.

## Data Availability

The data presented in this study is available upon request from the corresponding author.
